# Brainwave Self-Regulation During Bispectral Index^TM^ Neurofeedback in Trauma Center Nurses and Physicians After Receiving Mindfulness Instructions

**DOI:** 10.3389/fpsyg.2019.02153

**Published:** 2019-09-26

**Authors:** C. Michael Dunham, Amanda L. Burger, Barbara M. Hileman, Elisha A. Chance, Amy E. Hutchinson, Chander M. Kohli, Lori DeNiro, Jill M. Tall, Paul Lisko

**Affiliations:** ^1^Trauma, Critical Care, and General Surgery Services, St. Elizabeth Youngstown Hospital, Youngstown, OH, United States; ^2^Behavioral Medicine, St. Elizabeth Family Medicine Residency, Youngstown, OH, United States; ^3^Trauma and Neuroscience Research Department, St. Elizabeth Youngstown Hospital, Youngstown, OH, United States; ^4^Department of Anesthesiology, St. Elizabeth Youngstown Hospital, Youngstown, OH, United States; ^5^Department of Neurosurgery, St. Elizabeth Youngstown Hospital, Youngstown, OH, United States; ^6^Department of Nursing, St. Elizabeth Youngstown Hospital, Youngstown, OH, United States; ^7^Department of Biological Sciences, Youngstown State University, Youngstown, OH, United States; ^8^Pastoral Services, St. Charles Borromeo Catholic Church, Boardman, OH, United States

**Keywords:** Bispectral Index, BIS monitor, neurofeedback, stress, mindfulness, physicians, nurses

## Abstract

Fifty-seven level I trauma center nurses/physicians participated in a 4-day intervention to learn relaxed alertness using mindfulness-based instructions and EEG neurofeedback. Neurofeedback was provided by a Bispectral Index^TM^ (BIS) system that continuously displays a BIS value (0–100) on the monitor screen. Reductions in the BIS value indicate that power in a high-frequency band (30–47 Hz) is decreased and power in an intermediate band (11–20 Hz) is increased. A wellbeing tool with four positive affect and seven negative affect items based on a 5-category Likert scale was used. The wellbeing score is the sum of the positive affect items (positive affect score) and the reverse-scored negative affect items (non-stress score). Of functional concern were four negative affect items rated as moderately, quite a bit, or extremely in a large percent. Of greater concern were all four positive affect items rated as very slightly or none at all, a little, or moderately in over half of the participants. Mean and nadir BIS values were markedly decreased during neurofeedback when compared to baseline values. Post-session relaxation scores were higher than pre-session relaxation scores. Post-session relaxation scores had an inverse relationship with mean and nadir BIS values. Mean and nadir BIS values were inversely associated with NFB cognitive states (i.e., widening the visual field, decreasing effort, attention to space, and relaxed alertness). For all participants, the wellbeing score was higher on day 4 than on day 1. Participants had a higher wellbeing score on day 4 than a larger group of nurses/physicians who did not participate in the BIS neurofeedback trial. Eighty percent of participants demonstrated an improvement in the positive affect or non-stress score on day 4, when compared to day 1; the wellbeing, non-stress, and positive affect scores were substantially higher on day 4 than on day 1. Additionally, for that 80% of participants, the improvements in wellbeing and non-stress were associated with reductions in day 3 BIS values. These findings indicate that trauma center nurses/physicians participating in an EEG neurofeedback trial with mindfulness instructions had improvements in wellbeing.

**Clinical Trial Registration:** www.ClinicalTrials.gov, identifier NCT03152331. Registered May 15, 2017.

## Introduction

For nurses and physicians, concerns exist relative to emotional exhaustion, burnout, and job dissatisfaction. In a study of medical students, residents/fellows, and early career physicians, adverse manifestation rates were 30–40% for emotional exhaustion, 40–50% for burnout, 40–60% for depression, 7–9% for suicidal ideation, and 50–60% for fatigue (Dyrbye et al., 2014). Similar findings were noted in another investigation assessing matriculating medical students where the burnout rate was 27% and the depression rate was 26% (Brazeau et al., 2014). Also of concern is an investigation of practicing physicians that found the following rates: 31% for emotional hardening; 55% for burnout; 33% for depression; 10% for suicidal ideation; and 48% for fatigue (Dyrbye et al., 2013). The rate of emotional exhaustion, a risk for burnout, has been found to be substantial in nurses in the United States. Two studies have found mean hospital-based nurse emotional exhaustion scores of 24.5 and 24.3, indicating that the magnitude of this emotional exhaustion was present to a moderate degree (Vahey et al., 2004; Poghosyan et al., 2010). The United States emotional exhaustion scores are slightly higher than that found for a Swedish cohort (21) (Lindqvist et al., 2015). Surveys have indicated that 20–35% of hospital-based nurses have expressed the intent to leave their current job in the near future (Vahey et al., 2004; Chang et al., 2007; Lindqvist et al., 2015).

We have been unable to find a single study that includes United States physicians and nurses and then provides subset analyses that compare physician to nurse burnout in the same healthcare environment. However, we can make limited assessments based on separate studies. The 33% burnout proportion in a single intensive care unit nurse study (Poncet et al., 2007) is lower than the 40–55% proportion described in three physician intensive care and non-intensive care unit investigations (Embriaco et al., 2007; Dyrbye et al., 2013, 2014). Data from a systematic review of intensive care unit professionals (Chuang et al., 2016) indicates that the emotional exhaustion proportion for nurses has a mean of 32% (5 studies) and for physicians is 25% (1 study). These emotional exhaustion proportions are similar to those presented by Dyrbye et al. (2014) for non-intensive care unit physicians (30–40%). Non-intensive care unit physician emotional exhaustion scores provided by Dyrbye et al. (2014) (22–25) are similar to the 2 studies describing non-intensive care unit nurse emotional exhaustion scores (24) (Vahey et al., 2004; Poghosyan et al., 2010). The proportions of depression for non-intensive care unit physicians is reported to be 33–60% (Dyrbye et al., 2013, 2014), while the depression proportion for intensive care unit nurses has been found to be 30% (Mealer et al., 2007). The job dissatisfaction proportion for non-intensive care unit physicians has been reported to be 25% (Dyrbye et al., 2013) and 20–35% for non-intensive care unit nurses (Vahey et al., 2004; Chang et al., 2007; Lindqvist et al., 2015). We found 3 recent Middle Eastern studies demonstrating that physician and nurse burnout proportions and high emotional exhaustion proportions were similar when the physicians and nurses worked in the same healthcare environment (Abdo et al., 2016; Hamdan and Hamra, 2017; Alqahtani et al., 2019). Although burnout nurse and physician comparisons appear to be relatively similar, precise United States literature comparisons are mitigated by the lack of a study that describes both cohorts in the same environment using the same methodology.

### Neurofeedback

Neurofeedback (NFB) has been demonstrated to be potentially useful for decreasing anxiety (Michael et al., 2005; Dias and van Deusen, 2011; Cheon et al., 2015) and mitigating manifestations of alcohol use disorder (Dalkner et al., 2017), tinnitus (Hartmann et al., 2011; Weisz et al., 2011), and substance abuse (Unterrainer et al., 2013). Other investigations provided evidence that NFB enhances attention (Egner and Gruzelier, 2004; Wang and Hsieh, 2013), mood (Raymond et al., 2005), memory (Escolano et al., 2011), musical performance (Egner and Gruzelier, 2003), and surgical technique (Ros et al., 2009). NFB is a process in which an individual learns to intentionally alter their brainwave activity (Marzbani et al., 2016).

Although NFB has been performed using many diverse protocols, the following is a description of a commonly used procedure. Sensors are applied to an individual, and an electroencephalography (EEG) device computes the power (square of the microvolt measurement) of frequency bandwidths, according to cycles per second or Hertz (Hz), as follows: delta (0.5–4.0 Hz), theta (4–8 Hz), alpha (8–12 Hz), beta (12–30 Hz), and gamma (>30 Hz) (Marzbani et al., 2016). Commonly, a reward bandwidth target is chosen by the trainer, such as the sensorimotor rhythm at 12–15 Hz, which is an intermediate range associated with relaxed alertness (Gruzelier, 2014; Marzbani et al., 2016). Additionally, by using non-reward target ranges often set at 2–6 Hz and 22–36 Hz, a computer monitor screen produces visual symbolic images as a mechanism for providing moment-to-moment feedback to the trainee. The visual symbolic images change according to whether the bandwidth power is being increased within the reward or non-reward targets at any given moment, thus providing the trainee with a simplified pictorial of symbolic image representations that correlate with brainwave power functions. An individual is given instructions regarding desirable and undesirable visual display feedback image signals and is encouraged to maintain a state that is associated with the desirable brainwave target.

Over the past 10 years, a few studies have described EEG manifestations and post-NFB outcomes in nurses and physicians. Lees published a study in 2016 describing power spectral density estimates according to various EEG band frequencies in a cohort of nurses (Lees et al., 2016). Following stress induction, several band frequency power estimates significantly changed and were associated with cognitive performance, thus demonstrating that cognitive functionality can be expressed according to variations in brainwave activities. A recent study by Kelm et al. (2018) documented that NFB training enhanced performance during cardiac arrest simulations in healthcare providers. The cardiac arrest management teams included physicians, nurses, respiratory therapists, and pharmacists who underwent a 4-week period of NFB training. Following NFB training, cardiac arrest simulations demonstrated that the timeliness of performing critical patient care interventions and inter-personal teaming activities significantly improved. Another recent investigation by Nazer et al. (2018) assessed medical students who had undergone NFB training over a 2 month period. Post-test verbal memory scores were significantly greater, when compared to pre-test values in the NFB training group, yet there were no changes in the control cohort. In an older study by Ros et al. (2009), trainee ophthalmic microsurgeons undergoing NFB training, 8 sessions for 30 min each, were compared to surgical trainees without NFB in a simulation laboratory environment. Post-NFB training demonstrated significant score improvements in surgical skills and reductions in daily anxiety, when compared to pre-NFB values.

### Mindfulness

Mindfulness training has been associated with reductions in stress or burnout risk in literature reviews focusing on nurses (Smith, 2014) or physicians (Regehr et al., 2014) and in studies that include nurses and physicians (Goodman and Schorling, 2012; Kemper and Khirallah, 2015). Mindfulness is an attitudinal expression of receptive awareness, wherein there is a distinction made between an experience occurring in the present moment and associated thoughts and interpretations about that experience. The thinking process itself is observed with all thoughts being treated as equal in value, without attraction or rejection (Perlman et al., 2010). Of importance, mindfulness training can rely on attentional and visio-spatial components, and on cognitive abilities that can change according to the style of each subject, e.g., visualizer vs. verbalizer (Oliveri et al., 2012; Ardila and Rosselli, 2018).

Several attentional factors have been described in the literature that might also influence mindfulness. Narrow attentional focus is visual convergence, where specific, central external stimuli are volitionally selected, often with intense concentration, while peripheral visual objects are deselected (Fehmi and Shor, 2013; Sun et al., 2016). Widening the visual scope of attentional focus has been associated with relaxed attention and a balanced state of arousal and sympathetic and parasympathetic neural function (Fehmi and Shor, 2013). Of relevance, evidence has indicated that increasing the scope of the visual field enhances alpha brain wave activity with an attendant relaxed state of alertness (Plotkin, 1976; Fehmi and Shor, 2013). Narrow-focus attention has also been linked to increased fast brain wave intensity (increased amplitude in the beta-2 and beta-3 frequency bands) (Fehmi and Shor, 2013). Literature provides evidence that enhancing one’s awareness of external space positively influences brainwave activity (Plotkin, 1976; Fehmi and Shor, 2013; Hinterberger et al., 2014) and enhances relaxation (Birnbaum, 1978). The nursing literature includes advocacy for fostering external space awareness as a mechanism for advancing compassionate care and wholeness (Cowling et al., 2008; Jonas-Simpson, 2010).

Because of this evidence, the authors created specific mindfulness instructions (suggestions and prompts) that appeared likely to foster learning brainwave self-regulation in our research. The mindfulness instructions were assessed and found to be efficacious in our previous pilot study (Dunham et al., 2018). That is, there were substantial reductions in NFB BIS values, when compared to baseline, indicating that brainwave self-regulation was occurring following these mindfulness instructions.

### Combining Neurofeedback and Mindfulness

In 2013, an opinion article advocated combining mindfulness and NFB as a means for investigating conscious experiences and interactions between the mind and body (Brandmeyer and Delorme, 2013). NFB would appear to be a reasonable method for learning mindfulness, because mindfulness training can be influenced by attentional and visio-spatial components and on cognitive abilities that can change according to the style of each individual (Oliveri et al., 2012; Ardila and Rosselli, 2018). Following the recommendation for combining EEG NFB and mindfulness (Brandmeyer and Delorme, 2013), 4 relevant investigations have been published from 2016 to 2018. Of two investigations demonstrating an association between EEG NFB and mindfulness, one showed a link between NFB and effortless awareness (van Lutterveld et al., 2017), and the other showed an association between mindfulness and an ability to up-regulate sensorimotor rhythm power (Wood and Kober, 2018). The other two studies demonstrate that EEG NFB, in conjunction with mindfulness, improve subjective wellbeing (Bhayee et al., 2016) and decrease stress and anxiety (Balconi et al., 2018).

In 2017, a pilot study was formulated by the current authors and conducted to teach nurses and physicians how to enter into a state of relaxed alertness (receptive awareness) using BIS NFB (Dunham et al., 2018). To facilitate learning relaxed alertness, participants were provided with mindfulness instructions that might facilitate alterations in attention (cognitive states) during NFB. In that study, 10 nurse/physician participants underwent 21 learning days. One participant completed 3 learning days and another participated in 4 learning days. Both participants had substantial improvements in their wellbeing scores on days 3 and 4 respectively. Further, it was clear from the statistical analyses that the BIS values substantially decreased during NFB after the subjects were given the formulated mindfulness instructions, indicating that brainwave self-regulation had occurred. On the basis of these findings, the authors re-designed the study by adding one new item to the wellbeing tool, a survey that has been validated recently (Dunham et al., 2019), and establishing a compensation process to incentivize each participant to take part in 4 learning days. We also felt that we had sufficient evidence to continue using the same mindfulness instructions.

### Study Aim

The principal objective of the current study was to determine if NFB participants could learn to self-regulate their brainwave activities immediately after receiving mindfulness instructions. We also sought to determine if the subjects would have improvements in their wellbeing scores on day 4, when compared to day 1. Additionally, we wanted to determine if NFB (brainwave self-regulation) was associated with relaxation by having the subjects rate their state of relaxation before and after NFB. Because we wanted to find out if participants were aware of their state of attention or cognition when brainwave activity changed, we also had subjects rate each NFB cognitive state according to their perceptions that the state was associated with reductions in the BIS values.

## Materials and Methods

### Ethics Statement and Subjects

The St. Elizabeth Youngstown Hospital, Mercy Health Youngstown, LLC institutional review board (IORG# 0001624) approved the BIS NFB study and required signed, informed consent (approval number: 17-006; June 20, 2018). The institutional review board approved the completion of a long-term follow-up survey for participants in the BIS NFB study (approval number: 18-027; August 16, 2018). The institutional review board approved completion of the 11-item Wellbeing Inventory by physicians and nurses, without a requirement to participate in the BIS NFB study, and waived the need for consent (approval number: 18-031; September 27, 2018). Permission from the right’s holders of the monitor was not required to publish our data results.

**FIGURE 1:**
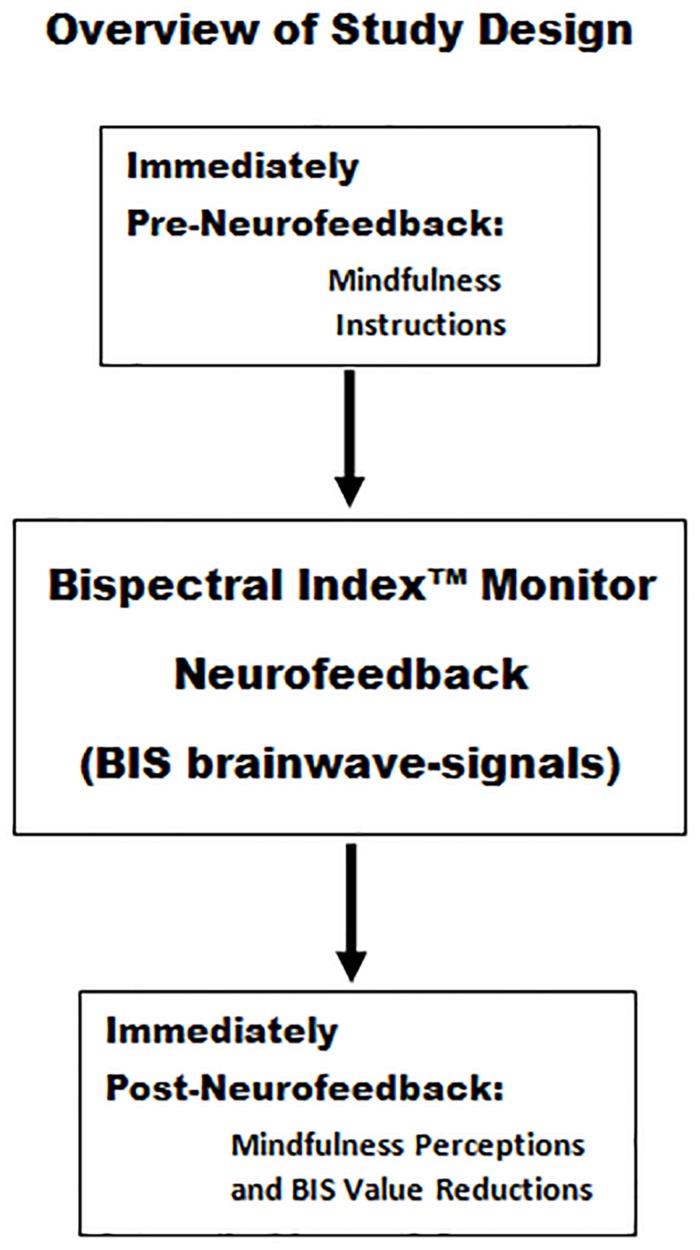
Mindfulness instructions were provided immediately prior to Bispectral Index^TM^ neurofeedback session 1 and session 2. Immediately following session 2, there was a survey assessment of the relationship between the subject’s employment of mindfulness instructions and their perceptions as to whether they were associated with reductions in BIS values.

**FIGURE 2:**
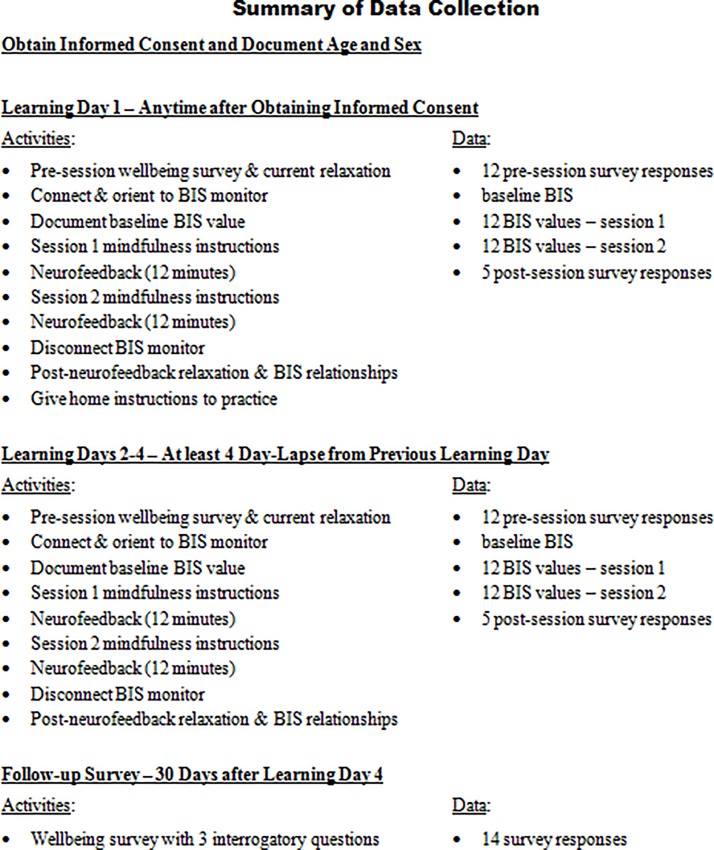
Key events and relevant data.

Physicians (resident, attending, and assistant) and nurses (registered, practitioner, and anesthetist), employed by St. Elizabeth Youngstown Hospital Level I Trauma Center, were welcomed to participate in the learning sessions. Exclusion criteria were individuals undergoing psychological or psychiatric counseling or those requiring psychiatric medications. Participants were compensated and encouraged to partake in 4 learning days with a minimum of 96 h between each learning day. Participants were also encouraged to complete the fourth learning day within 30 days of the first day. During the informed consent process, each subject was provided with an overview of the study design as summarized in Figures 1,  2.

### Pre-session Assessments

Before session 1 on each learning day, an 11-item St. Elizabeth Youngstown Hospital Wellbeing Inventory was completed by each participant (Dunham et al., 2019). The selection of the items included in the Wellbeing Inventory and the rationale for creating a new survey tool for hospital-based nurses and physicians undergoing NFB are described in the publication that demonstrates its validity (Dunham et al., 2019). The participants assessed their wellbeing over the most recent 3 days. The 4 positive affect items (restful sleep, energetic, alert, and enthusiastic) were each ranked as (1) very slightly or none at all, (2) a little, (3) moderately, (4) quite a bit, or (5) extremely. The positive affect score was the sum of the ratings for these 4 items. The 7 negative affect items (irritation, nervousness, overreaction, tension, feeling overwhelmed, feeling that people were too demanding, and feeling drained) were each ranked as (5) very slightly or none at all, (4) a little, (3) moderately, (2) quite a bit, or (1) extremely. The non-stress score was the sum of the reverse-scored negative affect ratings for the 7 items.

The wellbeing score was the sum of the positive affect and non-stress scores. The 11-item BIS NFB participant Wellbeing Inventory results were compared to data using the same survey, collected for survey validation, but completed by a cohort of nurses and physicians who did not participate in the BIS NFB study (referred to as non-participants) (Dunham et al., 2019). In addition to the 11-item Wellbeing Inventory, the BIS NFB participant’s current state of relaxation was rated as (1) very slightly or none at all, (2) a little, (3) moderately, (4) quite a bit, or (5) extremely.

By using a subset of the 11-item Wellbeing Inventory, an abbreviated, Non-burnout Inventory, was also used for participant assessments (Dunham et al., 2019). Two positive affect items (energetic and enthusiasm) and 3 negative affect or non-stress items (feeling overwhelmed, feeling that people were too demanding, and drained) were included. The non-burnout score was the sum of the 2 positive affect items and the 3 non-stress items. The Non-burnout Inventory score is a continuous variable where the 3 negative affect items are reverse coded. The score range is 5–25 with a higher score indicating a lower risk for burnout.

### BIS NFB Sessions

According to the manufacturer’s instructions, the BIS sensor was applied to the participant’s left or right forehead and temporal fossa at the discretion of the trainer. Each learning day consisted of two 12-min BIS NFB sessions, where each session was preceded by participant mindfulness instructions. Prior to providing session 1 instructions, participants were oriented to the BIS monitor screen, and a baseline BIS value was recorded. The session instructor documented the lowest BIS value observed during each session minute.

Just before session 1, the following mindfulness instructions were given, verbatim: “Sit comfortably with your hands on your legs. Keep your eyes open during the session. View your brainwave activity in the EEG display (trainer pointed to the raw EEG display). A BIS value more than 94 indicates fast brainwave activity, wherein attention might be associated with stress. Note that the BIS number might decrease by balancing relaxation with attention. Be curious to explore the feeling of relaxed attention.” Immediately after receiving these instructions, the first 12-min BIS NFB session began. A 1- to 2-min break was given between session 1 and session 2.

Just before session 2 began, the following additional mindfulness instructions were given, verbatim: “Listen to these suggestions that might foster relaxed attention. Do not think too intensely. If thoughts arise, gently return your attention to the activity. Try not to focus too narrowly on the BIS number and use modest relaxation. Simply observe the BIS number, accepting each value as it is. In addition to looking at the BIS number, be aware of the entire monitor display. Also, notice the space to the left of the monitor. Additionally, notice the space to the right of the monitor. Gently, be aware of the spaces to the left and right of the monitor.” Immediately after receiving these instructions, the second 12-min BIS NFB session began.

### Post-session Assessments

At the conclusion of session 2 on each learning day, the participants were asked to subjectively rate NFB cognitive states that seemed to have influenced reductions in BIS values during sessions 1 and 2. The NFB cognitive states rated were (a) widening the visual field, (b) decreasing effort, (c) attention to space, and (d) relaxed alertness. Each NFB cognitive state was rated as (1) very slightly or none at all, (2) a little, (3) moderately, (4) quite a bit, or (5) extremely. Following session 2, the participant was also asked to rate their current state of relaxation, which was rated as (1) very slightly or none at all, (2) a little, (3) moderately, (4) quite a bit, or (5) extremely.

On only learning day 1, after the post-session surveys, participants were given practice mindfulness instructions to apply during everyday activities. The practice instructions were as follows: “Periodically, apply the following guidelines when you look at objects or undertake activities (e.g., reading a computer screen, watching television, and vacuuming). Be curious to explore the feeling of relaxed attention. Do not think too intensely. Do not focus too narrowly on the object or activity; widen your field of vision instead. Simply observe the object or activity, accepting it as it is. Notice the space to the left of the object or activity. Notice the space to the right of the object or activity. Gently, be aware of the space. If thoughts arise, gently return your attention to your object or activity.”

### Long-Term Follow-Up Survey

Each participant was contacted to complete another 11-item Wellbeing Inventory survey approximately 30 days after learning day 4. In addition to the wellbeing inventory, participants were asked the following three interrogatory (yes or no) questions: (1) Since your last learning day, have you made an effort to apply what you learned in your daily activities? (2) Overall, do you believe that your wellbeing has benefited from learning receptive awareness? (3) Did your participation stimulate interest in methods of improving your wellbeing? The authors made two attempts to contact BIS NFB participants to complete the survey.

### Statistical Analyses

Results were entered into an Excel 2010 worksheet (Microsoft Corp., Redmond, WA, United States) and imported into the SAS System for Windows, release 9.2 (SAS Institute Inc., Cary, NC, United States). All mean values are accompanied by their standard deviations. Two-group proportion comparisons were assessed using the Fisher exact test. For two-group ordinal rank data comparisons among the same participants, the mean and standard deviation, paired *t*-test result, Cohen *d*, median, range, and paired Wilcoxon (signed-rank) test result were assessed. For comparisons with greater than 2 groups, PROC ANOVA in SAS was used. For ordinal ranked data comparisons between groups with different participants, the Wilcoxon rank-sum test was used. Multivariate regression assessments were performed using PROC REG in SAS. Correlation analyses were assessed in SAS using Spearman rank-order procedures. The level of significance was *p* < 0.05.

## Results

### BIS NFB Participants and Control Group

From July 2018 to October 2018, 57 trauma center nurses/physicians participated in 4 learning days each, totaling 228 days. Of these BIS NFB participants, there were 11 men (19.3%) and 46 women (80.7%), and their mean age was 36.5 ± 11.8 years (range 24–67 years). A control group consisted of 191 nurses/physicians from the same trauma center who did not participate in the BIS NFB investigation, but completed the same 11-item Wellbeing Inventory survey (Dunham et al., 2019).

### BIS NFB Value Reductions

Baseline BIS was recorded for all 228 learning days. The 5,439 BIS NFB values were used to compute a mean BIS NFB value for each of the 228 learning days. Baseline, mean, and nadir BIS values were similar on days 1, 2, 3, and 4 (see Table 1).

**TABLE 1:** BIS values and wellbeing scores for each learning day.

	**Day 1**	**Day 2**	**Day 3**	**Day 4**	
	***n* = 57**	***n* = 57**	***n* = 57**	***n* = 57**	**ANOVA**
Baseline BIS	97.4 ± 0.8	97.6 ± 0.8	97.6 ± 0.7	97.5 ± 0.7	*p* = 0.2651
Mean BIS	90.4 ± 4.2	90.7 ± 4.4	90.2 ± 4.6	90.0 ± 5.0	*p* = 0.8537
Nadir BIS	84.0 ± 6.0	84.1 ± 4.9	83.7 ± 6.6	83.8 ± 7.2	*p* = 0.9811
					**Paired *t*-test**
Wellbeing Score	38.8 ± 5.9	40.1 ± 6.1	41.1 ± 5.4	41.3 ± 5.7	*p* = 0.0056
Non-stress Score	27.1 ± 4.6	28.4 ± 4.9	29.3 ± 4.2	29.6 ± 4.3	*p* = 0.0008
Positive Affect Score	11.7 ± 2.7	11.6 ± 2.8	11.8 ± 2.9	11.8 ± 2.7	*p* = 0.6251
Non-burnout Score	16.8 ± 3.4	17.7 ± 3.3	18.1 ± 2.9	18.3 ± 2.8	*p* = 0.0036

*BIS, Bispectral Index^*TM*^; ANOVA, analysis of variance. Each day, the mean BIS and nadir BIS were lower than the baseline BIS (*p* < 0.0001). Paired *t*-tests are comparisons of day 1 and day 4 values.*

The 228 mean BIS NFB values were lower (90.3 ± 4.5) than the 228 BIS baseline values (97.5 ± 0.8; paired *t*-test *p* < 0.0001; Cohen *d* 2.2). The 228 nadir BIS NFB values were lower (83.9 ± 6.2) than the 228 BIS baseline values (97.5 ± 0.8; paired *t*-test *p* < 0.0001; Cohen *d* 3.1). The distributions of the mean and nadir BIS NFB values are shown in Table 2.

**TABLE 2:** Distributions of 228 mean and nadir BIS NFB values.

**Mean BIS**		
79–90	109	47.8%
91–95	89	39.0%
96–98	30	13.2%
**Nadir BIS**		
55–79	32	14.0%
80–85	113	49.6%
86–92	70	30.7%
93–98	13	5.7%

*BIS, Bispectral Index^*TM*^; NFB, neurofeedback.*

For the 57 participants on day 1, the mean BIS NFB values were lower than the BIS baseline values (paired *t*-test *p* < 0.0001; Cohen *d* 1.7) as were the nadir BIS NFB values (paired *t*-test *p* < 0.0001; Cohen *d* 3.1) (see Table 1). In the same 57 participants on day 1, the session 2 BIS NFB values were lower (89.6 ± 6.0) than the session 1 values (91.3 ± 5.2; *p* < 0.0001). For the 57 participants on day 1, the proportion of BIS NFB values that were ≤95 was larger for session 2 (528 [78.6%]) than for session 1 (491 [72.0%]; *p* = 0.0056; odds ratio [OR] 1.4).

### BIS NFB Value Associations

Age had a significant negative association with the mean BIS NFB values (*r* = −0.19; *p* = 0.0051) but no association with the nadir BIS NFB values (*r* = −0.08; *p* = 0.2378). For the 228 learning days, the post-session relaxation scores were higher (3.6 ± 0.8) than the pre-session relaxation scores (2.7 ± 0.8; paired *t*-test *p* < 0.0001; Cohen *d* 1.0; paired Wilcoxon *p* < 0.0001). For the 228 learning days, the post-session relaxation scores had an inverse relationship with the nadir BIS NFB values (*r* = −0.26; *p* < 0.0001) and mean BIS NFB values (*r* = −0.23; *p* < 0.0001).

Post-session subjective ratings for NFB cognitive states that were perceived to influence BIS NFB reductions (widening visual field, decreasing effort, attention to space, and relaxed alertness) each had a median of 3.0 (range 1–5). The nadir and mean BIS NFB values had inverse associations with each of the four subjective ratings (see Table 3). Multivariate regression analysis demonstrated that nadir NFB BIS values had an independent association with relaxed alertness (*p* < 0.0001) and widening the visual field (*p* = 0.0022) and that mean NFB BIS values had an independent association with relaxed alertness (*p* < 0.0001) and decreasing effort (*p* = 0.0067).

**TABLE 3:** Subjective impression ratings that a NFB cognitive state was helpful in decreasing the BIS value and their associations with objectively measured nadir and mean BIS NFB values.

	**Nadir NFB BIS**		**Mean NFB BIS**	
		
**Subjective Ratings**	***r*-value**	***p*-value**	***r*-value**	***p*-value**
Widening Visual Field	−0.35	<0.0001	−0.24	0.0003
Decreasing Effort	−0.32	<0.0001	−0.41	<0.0001
Attention to Space	−0.26	<0.0001	−0.17	0.0095
Relaxing	−0.42	<0.0001	−0.47	<0.0001

*BIS, Bispectral Index^*TM*^; NFB, neurofeedback.*

### BIS NFB Participants and Wellbeing Inventory Survey Responses

Of the 114 11-item Wellbeing Inventory surveys completed on days 1 and 2 of the BIS NFB study, the seven negative affect items were rated moderately to extremely with a range of 13–41% (see Table 4). Half of the surveys showed that ≥2 of 7 items were rated moderately to extremely (see Table 4). The four positive affect items were rated very slightly or none at all to moderately with a range of 60–80% (see Table 5). Seventy-five percent of the surveys showed that ≥3 of 4 items were rated very slightly or none at all to moderately (see Table 5). For the 228 learning days, the wellbeing score had a significant association with the pre-session relaxation score (*r* = 0.46; *p* < 0.0001).

**TABLE 4:** Negative affect item results for learning days 1 and 2 (*n* = 114).

**Negative Affect Items**	**Moderately-to-Extremely**
Irritation	34 (29.8%)
Nervousness	30 (26.3%)
Overreaction	15 (13.2%)
Tension	47 (41.2%)
Feeling Overwhelmed	45 (39.5%)
People Too Demanding	30 (26.3%)
Feeling Drained	30 (26.3%)
0 of 7 Items	38 (33.3%)
≥1 of 7 Items	76 (66.7%)
≥2 of 7 Items	60 (52.6%)
≥3 of 7 Items	42 (36.8%)

**TABLE 5:** Positive affect item results for learning days 1 and 2 (*n* = 114).

**Positive Affect Items**	**Very Slightly-to-Moderately**
Restful Sleep	90(79.0%)
Energetic	91(79.8%)
Alert	71(62.3%)
Enthusiastic	91(79.8%)
0 of 4 Items	6(5.3%)
≥1 of 4 Items	108(94.7%)
≥2 of 4 Items	96(84.2%)
≥3 of 4 Items	86(75.4%)

### Wellbeing Scores on Day 4

For the 57 participants, the 11-item wellbeing score was higher on day 4, when compared to day 1 (paired *t*-test *p* = 0.0056; paired Wilcoxon *p* = 0.0067) (see Table 1). The non-stress and non-burnout scores were also higher on day 4, when compared to day 1 (see Table 1). Of the 57 participants, 13 (22.8%) failed to show an improvement in the positive affect or non-stress score on day 4, when compared to day 1. Of the 13 BIS NFB participants who did not have an improvement in either the positive affect or non-stress score on day 4, 6 participants had a day 1 wellbeing score ≥ 44.

Among the 57 participants, 44 (77.2%; [95% confidence interval 64.8–86.2%]) demonstrated an improvement in the positive affect or non-stress score on day 4, when compared to day 1. Of the 44 participants with improved scores, 28 (63.6%) had their greatest improvement with the non-stress score. In these 28, the non-stress score was higher on day 4 (30.8 ± 3.2), when compared to day 1 (25.0 ± 4.6; paired *t*-test *p* < 0.0001; Cohen *d* 1.5; paired Wilcoxon *p* < 0.0001). Of the 44 participants with improved scores, 16 (36.4%) had their greatest improvement with the positive affect score. In these 16, the positive affect score was higher on day 4 (13.1 ± 2.3), when compared to day 1 (10.6 ± 2.5; paired *t*-test *p* < 0.0001; Cohen *d* 1.0; paired Wilcoxon *p* < 0.0001). For all 44 participants with an improved score on day 4, the wellbeing, non-stress, positive affect, and non-burnout scores were significantly higher on day 4 than on day 1 (see Table 6).

**TABLE 6:** Forty four subjects with improved non-stress score or positive affect score on day 4.

	**Day 1**	**Day 4**	**Paired *t*-test *p***	**Paired Wilcoxon *p***	**Cohen *d***
Wellbeing Score	37.9 ± 5.7	42.6 ± 4.8	<0.0001	<0.0001	0.9
Non-stress Score	26.7 ± 4.7	30.9 ± 3.3	<0.0001	<0.0001	1.0
Positive Affect Score	11.2 ± 2.7	12.1 ± 2.5	0.0060	0.0096	0.4
Non-burnout Score	16.2 ± 3.2	18.9 ± 2.3	<0.0001	0.0001	1.0

### Wellbeing Improvements and BIS Associations

For all 44 participants with an improved score on day 4, the proportion of minutes with a BIS NFB value ≤95 was greater on day 4 (790 [75.2%]) than on day 1 plus day 2 (1,472 [70.4%]; *p* = 0.0052; OR 1.3). For all 44 participants, day 3 BIS NFB values were dichotomized as ≤95 or ≥96 and nadir values were dichotomized as <90 or ≥90. Lower day 3 BIS dichotomous values were associated with greater increases in day 4 minus day 1 wellbeing scores and non-stress scores (see Table 7).

**TABLE 7:** Associations of day 4 minus day 1 wellbeing scores and non-stress scores with day 3 BIS values in 44 subjects with improved non-stress or positive affect scores on day 4.

**Each Day 3 BIS Value**	**Minutes**	**Wellbeing Difference**	**Wilcoxon *p***
**Day 4 Wellbeing Score minus Day 1 Wellbeing Score**			
≤95	755	5.0 ± 4.9	
≥96	296	4.2 ± 5.4	0.0189

**Each Day 3 BIS Nadir**	**Subjects**	**Wellbeing Difference**	**Wilcoxon *p***

<90	35	5.5 ± 4.7	
≥90	9	1.8 ± 5.6	0.0466

**Each Day 3 BIS Value**	**Minutes**	**Non-stress Difference**	**Wilcoxon *p***

**Day 4 Non-stress Score minus Day 1 Non-stress Score**			
≤95	755	4.4 ± 4.3	
≥96	296	2.9 ± 4.9	<0.0001

**Each Day 3 BIS Nadir**	**Subjects**	**Non-stress Difference**	**Wilcoxon *p***

<90	35	4.7 ± 4.1	
≥90	9	1.1 ± 5.5	0.0327

*BIS, Bispectral Index.^*TM*^*

### BIS NBF Participants Compared to Control Group

Nurses/physicians not participating in the BIS NFB study (control group) completed the 11-item Wellbeing Inventory (*n* = 191) (Dunham et al., 2019). The wellbeing scores were similar for the 191 subjects in the control group and the 57 BIS NFB participants on learning day 1 (see Table 8). On learning day 4, the wellbeing scores were significantly higher for the 57 BIS NFB participants than for the control group (see Table 8). The wellbeing scores were similar for the control group and the 44 improved-score BIS NFB participants on learning day 1 (see Table 8). On learning day 4, the wellbeing scores were significantly higher for the 44 improved-score BIS NFB participants than for the control group (see Table 8).

**TABLE 8:** Comparisons of wellbeing scores in BIS NFB subjects and control group.

	**Control Group**	**NFB – Day 1**	***p*-value**	**Cohen *d***
	***n* = 191**	***n* = 57**		
**Wellbeing Score Comparisons for Day 1 BIS NFB Subjects**				
Wellbeing Score	37.0 ± 7.5	38.8 ± 5.9	0.1074	0.3

	**Control Group**	**NFB – Day 4**	***p*-value**	**Cohen *d***
	***n* = 191**	***n* = 57**		

**Wellbeing Score Comparisons for Day 4 BIS NFB Subjects**				
Wellbeing Score	37.0 ± 7.5	41.3 ± 5.7	<0.0001	0.7

	**Control Group**	**NFB – Day 1**	***p*-value**	**Cohen *d***
	***n* = 191**	***n* = 44**		

**Wellbeing Score Comparisons for Day 1 BIS NFB (subjects with improved day 4 scores)**				
Wellbeing Score	37.0 ± 7.5	37.9 ± 5.7	0.4174	0.1

	**Control Group**	**NFB – Day 4**	***p*-value**	**Cohen *d***
	***n* = 191**	***n* = 44**		

**Wellbeing Score Comparisons for Day 4 BIS NFB (subjects with improved day 4 scores)**				
Wellbeing Score	37.0 ± 7.5	42.6 ± 4.8	<0.0001	0.9

*BIS, Bispectral Index^*TM*^; NFB, neurofeedback.*

### Follow-Up Surveys

Of the 24/57 (42.1%) follow-up survey respondents, 4 (16.7%) did not improve their non-stress or positive affect score on day 4. The 16.7% non-improvement rate in the respondents was similar to the overall non-improvement rate in the 57 participants (22.8% [13/57]; *p* = 0.7660). The mean days following their fourth learning day were 47 ± 14.6 (28–89). Three interrogatories were included with the survey. To the question, since the last learning day, have you made an effort to apply what you learned in your daily activities?, 21/24 (87.5%) of respondents replied with a yes. To the question, do you believe that your wellbeing has benefited from learning receptive awareness?, 22/24 (91.7%) of respondents replied with a yes. To the question, did your participation stimulate interest in methods of improving your wellbeing?, 23/24 (95.8%) of respondents replied with a yes. The proportion of respondents with an improvement in the wellbeing score from day 1 to day 4 was 18/24 (75.0%). Of these 18, the wellbeing score at follow-up was the same or higher in 8 (44.4%), when compared to that on day 4. The time from day 4 to follow-up for these 8 respondents was 54.6 ± 15.2 days (range 32–76). The proportion of respondents with an increased wellbeing score on the follow-up day, when compared to day 1, was 11/24 (45.8%). The proportion of respondents with an increased or same wellbeing score on the follow-up day, when compared to day 4, was 16/24 (66.7%).

## Discussion

### BIS NFB Value Reductions

The mean and nadir BIS NFB values from the 228 learning days were substantially reduced compared to baseline BIS values. This demonstrates the ability of the participants to self-regulate brainwave activity during the learning sessions in a compelling manner. Such findings were observed on learning day 1, implying that self-regulated brainwave alterations were quickly learned. The greater reductions in BIS NFB values during session 2 compared to session 1 suggest that learning is progressive with additional experience. It is also likely that the additional mindfulness instructions given before session 2 provide additional suggestions, and thus, opportunities and options for altering attention. The mean and nadir BIS NFB values did not substantially differ from day 1 to day 4, likely due to the prompt learning of self-regulated brainwave alterations on day 1.

The subjects understood that the goal during NFB was to decrease their BIS values, with the implication that they would be entering a state of relaxed alertness. The mindfulness instructions were prompts or suggestions provided to the subjects as attentional adjustments that could be employed during NFB as potential mechanisms for self-regulating brainwave activity. Brainwave self-regulation was documented to occur during the NFB sessions in that mean and nadir BIS values were substantially decreased, when compared to baseline values. Based on the extensive knowledge regarding the BIS monitor, it is reasonable to consider that a state of relaxed alertness exists when BIS values decrease (Glass et al., 1997; Zhong et al., 2005; Haberland et al., 2011). This view is also supported because subjects had perceptions of increased relaxation immediately following NFB.

### BIS NFB Value Associations

The mean BIS values had a significant inverse relationship with age. This finding suggests that younger participants were less successful in self-regulating brainwave activities or entering into a state of relaxed alertness. The post-session relaxation scores were higher than pre-session relaxation scores, indicating that the BIS NFB process was associated with an enhanced state of relaxation immediately after the intervention. The inverse association between the post-session relaxation scores and the nadir and mean BIS NFB values further link the degree of relaxation to the degree of self-regulation of brainwave activities.

Also of interest were the inverse associations among nadir and means BIS NFB values and the NFB cognitive states (widening the visual field, decreasing effort, attention to space, and relaxed alertness). The multivariate regression analysis suggests that relaxed alertness is the most apparent NFB cognitive state for self-regulating brainwave activities. Learning brainwave self-regulation and relaxed alertness was documented to have occurred, because the subjects’ perceptions that mindfulness prompts were associated with BIS value reductions was corroborated using correlation statistical analyses. That is, the subjects were able to pay attention to their style of attention and determine, in-the-moment, when those effects were associated with BIS reductions. In summary, these observations suggest that participants were able to associate styles of paying attention to actual changes in brainwave activities, fostering the notion that learning was indeed taking place.

### BIS Monitoring as a Model for NFB

The Food and Drug Administration classifies the BIS monitor as an EEG monitoring device that monitors EEG signals, and it may be used to monitor the effects of anesthetic and sedating agents. The credibility and validity of the BIS device for monitoring general anesthesia and conscious sedation are supported by more than 2,500 citations in the National Library of Medicine, which includes publications in the *New United Kingdom Journal of Medicine* (Avidan et al., 2008) and *Cochrane Systematic Review* (Punjasawadwong et al., 2014). Several studies have demonstrated significant associations between BIS monitor values (0–100) and clinical status with use of the Modified Observer’s Assessment of Alertness/Sedation Scale in patients undergoing general anesthesia (Glass et al., 1997; Zhong et al., 2005) or conscious sedation (Haberland et al., 2011).

Reductions in BIS values have also been found for conditions other than pharmacologic sedation and include acupressure (Fassoulaki et al., 2007), stage I sleep (Tung et al., 2002; Benini et al., 2005; Dahaba et al., 2011), guided imagery relaxation (Hudetz et al., 2004), restricted external stimulation therapy (flotation) (Dunham et al., 2017), and video relaxation (Tsutsumi et al., 2017). For BIS values between 60 and 100, the level highly correlates (*r* = 0.90; *p* < 0.01) with the ratio of power in a brainwave band with high frequency (30–47 Hz) relative to a lower frequency band (11–20 Hz) (Morimoto et al., 2004). That is, as the BIS value (the symbolic image being displayed) decreases, there is a relative linear reduction in the high frequency brainwave power relative to lower frequency brainwave power. In other words, BIS values are expressions of relative power in select frequency bandwidths. The user friendliness of the BIS monitor, the practical and reliable application of BIS sensors, and a single BIS value that represents the power of high-frequency band waves relative to the power of intermediate-frequency band waves facilitates data management are likely to appeal to other NFB investigators.

### Wellbeing Inventory Survey Responses

Of functional concern was the 4 negative affect items (tension, feeling overwhelmed, feeling that people were too demanding, and feeling drained) rated as moderately, quite a bit, or extremely by the trauma center nurses/physicians. Each of these negative affect items had an occurrence that ranged from 21 to 35%. Also of concern was the observation that 2 or more of the 7 negative affect items were rated as moderately, quite a bit, or extremely in 40% of the participants, and 3 or more of the negative affect items in 30%. Of greater concern were positive affect rankings, where restful sleep, energetic, alert, and enthusiastic items were rated as very slightly or none at all, a little, or moderately in 60–80% of participants. The pervasiveness of this finding is expressed by the observation that three-quarters of the participants had at least 3 of the 4 positive affect items rated as very slightly or none at all, a little, or moderately. These findings are consistent with other studies’ findings demonstrating that nurses and physicians have substantial burdens of emotional exhaustion, burnout, and job dissatisfaction that are associated with positive affect and negative affect (Agho, 1993; Vahey et al., 2004; Chang et al., 2007; Poghosyan et al., 2010; Dyrbye et al., 2013, 2014; Brazeau et al., 2014; Lindqvist et al., 2015; Dunham et al., 2019).

The 11-item Wellbeing Inventory survey has been validated, following the current study, according to psychometric properties and concurrent validity correlation with the Positive and Negative Affect Schedule (Dunham et al., 2019). It is of interest that the wellbeing score had a significant association with the pre-session relaxation score. That is, this finding provides additional evidence that the 11-item Wellbeing Inventory is a valid survey tool.

### Wellbeing Scores on Day 4

For the 57 nurse/physician participants, the 11-item Wellbeing Inventory score was higher on day 4 than on day 1, suggesting an overall benefit for those partaking in 4 BIS NFB days. Of the 57 participants, 20% failed to show an improvement in the positive affect or non-stress score on day 4 compared to day 1. Of the 13 participants who did not have an improvement in either the positive affect or non-stress score on day 4, six participants had a day 1 wellbeing score ≥44. Because the 11-item average rating would have been 4, it is improbable that these participants would have improved their day 4 score when compared to day 1. This suggests that approximately 10% of eligible nurse/physician candidates are unlikely to benefit from BIS NFB. If these 6 participants were removed from consideration, the observed improvement rates in positive affect or non-stress score would have increased to nearly 90%. Evidence also exists in the literature that some subjects do not seem to be able to learn brainwave self-regulation, as nearly one-third could not create alpha down-regulation (Nan et al., 2018).

Of the 44 participants with improved scores, 60% had their greatest improvement with the non-stress score, where non-stress score differences on day 4 were markedly higher than those on day 1. These findings indicate that irritation, nervousness, overreaction, tension, feeling overwhelmed, feeling that people were too demanding, and feeling drained were less of a problem on day 4. Of the 44 participants with improved scores, 40% had their greatest improvement with the positive affect score, where positive affect score differences on day 4 were substantially greater than those on day 1. These observations indicate that restful sleep and feeling more energetic, alert, and enthusiastic were experienced to a greater degree. These data representations provide an expression of maximal benefit according to positive affect and negative affect sub-domains, which have been recognized in the literature (Watson and Tellegen, 1985).

For all 44 participants with an improved positive affect or non-stress score on day 4, the wellbeing, non-stress, positive affect, and non-burnout scores were higher on day 4 than on day 1. The day 4 differences for the wellbeing, non-stress, and non-burnout scores were quite compelling, whereas the positive affect improvements were less dramatic, although still statistically significant. Positive affect has been recognized by other researchers as distinct from negative affect from a psychopathological standpoint and as fundamentally different processes (Watson and Tellegen, 1985). Further, compared to negative affect, positive affect has been found to better correlate with hospital-based nurses in regard to job satisfaction (Agho, 1993). The improvement in positive affect in this cohort is of potential importance, because Agho has demonstrated that job satisfaction in hospital-based nurses is associated with positive affect (Agho, 1993). Improvements in the non-burnout scores are potentially important, since hospital-based nurse studies demonstrate that emotional exhaustion is common (Vahey et al., 2004) and reducing burnout may be an effective strategy for improving quality of care (Poghosyan et al., 2010).

### Wellbeing Improvements and BIS Associations

For the participants with an improved positive affect or non-stress score on day 4, the proportion of minutes with a decreased BIS NFB value was greater on day 4 than on day 1 plus day 2. These data indicate that BIS NFB value reduction was progressive as the learning days increased, despite the substantial reduction in values that occurred on day 1. The improvement in the day 4 wellbeing score minus the day 1 wellbeing score difference was greater for the day 3 NFB BIS values that were reduced, when compared to values without a reduction. This finding demonstrates that improvements in day 4 wellbeing scores, relative to day 1, are related to lower day 3 BIS values or more effective brainwave alterations. It seems likely that day 3 values are likely to be, in part, a reflection of learning relaxed alertness on days 1 and 2 and obviously antedates the day 4 wellbeing scores. These data represent a linkage between the degree of day 3 BIS NFB alterations and the level of wellbeing improvements on day 4 as compared to day 1 values. Supportive results were also found in the relationship of day 3 nadir BIS NFB and day 4 wellbeing increments. The identification of associations between the day 3 BIS NFB decrements and improvements in the day 4 non-stress scores also supports such a relationship.

### BIS NFB Participants Compared to Control Group

The similarities of the wellbeing scores between the 57 day 1 BIS NFB participants and the control group suggest that the NFB participants were representative of a larger nurse/physician population. The higher wellbeing scores on day 4 of the 57 BIS NFB participants, when compared to the control group, suggest that the participants had improvements in wellbeing by participating in learning relaxed alertness (receptive awareness). Similar observations were made when only the 44 participants with day 4 improvements in positive affect or non-stress scores were assessed. These findings add supportive evidence that participation in the NFB sessions were beneficial to nurse/physician wellbeing.

### Follow-Up Surveys

The proportions of participants who failed to improve the non-stress or positive affect score on day 4 were similar for the follow-up survey respondents and the total group, suggesting that the respondents were representative of the parent group. Of the respondents, 90% indicated that they had and continued to benefit from learning receptive awareness, an experience that also stimulated their general interest in improving personal wellbeing. Certainly, these subjective testimonials are in harmony with the objective evidence provided by the study results, further indicating that participants experienced a benefit from the BIS NFB interventions.

Nearly 70% of survey respondents had an increased or same wellbeing score on the follow-up day, when compared to day 4, indicating that wellbeing was relatively durable and sustainable for well over a month following the NFB intervention. For the three-quarters of respondents with an improvement in wellbeing from day 1 to day 4, the follow-up wellbeing score nearly 2 months later was the same or higher as that on day 4 in 40%. These observations suggest that improvements in wellbeing with BIS NFB are preserved for a lengthy period in a substantial percentage of participants.

### Limitations

The biggest limitation of this study is that there was no wellbeing score follow-up for the cohort (191 nurses/physicians) that did not participate in the BIS NFB study. It would have been optimal to have a wellbeing survey completed approximately 3–4 weeks after the initial survey in the same non-participants to establish normal variances in nurse/physician wellbeing. If the subsequent wellbeing scores did not significantly increase, this would have provided additional evidence for a benefit with the BIS NFB intervention. Another limitation is the lack of more extensive epidemiological data for the BIS NFB participants. However, we believe that the minimal epidemiologic data, age and sex, likely enhance study recruitment by respecting participant privacy.

## Conclusion

The marked reductions in BIS values during NFB indicate that brainwave self-regulation was prominent and could be rapidly learned. The mindfulness instructions were prompts or suggestions provided to the subjects immediately prior to NFB as potential attentional adjustments that could be employed during NFB for altering their state of consciousness and self-regulating brainwave activity. The inverse associations of BIS reductions with ratings of their cognitive state following mindfulness instructions imply that the participants were simultaneously aware of brainwave alterations and their cognitive state. Therefore, this evidence suggests that participants were indeed paying attention to how they paid attention and learning brainwave self-regulation. Based on the extensive knowledge regarding the BIS monitor, it is reasonable to consider that a state of relaxed alertness exists when BIS values decrease. This view is also supported because subjects had perceptions of increased relaxation immediately following NFB. The survey responses indicate that the level I trauma center BIS NFB participants had concerning positive affect and negative affect findings. Multiple analyses in the current study indicate that participants had improvements in wellbeing as the BIS NFB interventions progressed. The association of the pre-session wellbeing and relaxation scores is an additional indication of validity in regard to the Wellbeing Inventory survey.

## Data Availability Statement

The datasets generated for this study are available on request to the corresponding author.

## Ethics Statement

The studies involving human participants were reviewed and approved by Institutional Review Board, Mercy Health Youngstown, LLC. The patients/participants provided their written informed consent to participate in this study.

## Author Contributions

CD, AB, BH, EC, AH, CK, LD, JT, and PL contributed to the conception and design of the study. BH and EC conducted the neurofeedback sessions and documented the data (informed consent, Bispectral Index^TM^ values, and all surveys). CD performed data entry and statistical analysis, and organized physician and nurse wellbeing data for those not participating in the neurofeedback study. CD, AB, BH, and EC reviewed the initial data results. CD wrote the first draft of the manuscript. All authors contributed to the manuscript revision, and read and approved the submitted version.

## Conflict of Interest

The authors declare that the research was conducted in the absence of any commercial or financial relationships that could be construed as a potential conflict of interest.
